# Twenty‐Four Hour Rest–Activity Rhythm Disturbances and Neural Alterations Associated With Emotion Regulation in Shift Workers

**DOI:** 10.1111/jsr.70052

**Published:** 2025-04-03

**Authors:** Kyung Hwa Lee, Ha Young Lee, Jeong Eun Jeon, Mi Hyun Lee, Jooyoung Lee, Jiyoon Shin, Min Cheol Seo, Yu Jin Lee, Seog Ju Kim

**Affiliations:** ^1^ Department of Psychiatry and Center for Sleep and Chronobiology Seoul National University, College of Medicine and Hospital Seoul Republic of Korea; ^2^ Division of Child and Adolescent Psychiatry, Department of Psychiatry Seoul National University Hospital Seoul Republic of Korea; ^3^ Seoul Top Class Clinic Seoul Republic of Korea; ^4^ Department of Psychiatry Sungkyunkwan University College of Medicine, Samsung Medical Center Seoul Republic of Korea; ^5^ Department of Psychiatry Seoul National University College of Medicine & SMG‐SNU Boramae Medical Center Seoul Republic of Korea; ^6^ Department of Psychiatry Veteran Health Service Medical Center Seoul Republic of Korea

**Keywords:** 24‐h rest‐activity rhythm, emotion regulation, functional magnetic resonance imaging, neural basis, shift work, sleep

## Abstract

We examined the neural basis of emotion regulation in shift workers, and the relationships between the neural basis of emotion regulation, mood, sleep disturbance and 24‐h rest–activity rhythm (RAR). Fifty‐six shift workers (SW) with non‐standard shift schedules and 52 controls (CON) participated in this study. They completed self‐reported measures of sleep and mood problems, kept a sleep diary, and wore a wrist actigraphy device to assess sleep and 24‐h RAR. They underwent one‐night polysomnography and were scanned while performing an emotion regulation task. We examined group differences in the neural basis of emotion regulation and correlations between neural, mood, sleep and 24‐h RAR variables. SW showed greater sleep disturbance (i.e., lower actigraphy‐estimated sleep efficiency) and altered 24‐h RAR (e.g., lower actigraphy‐estimated interdaily stability) than CON. SW also exhibited increased anterior insula (AI) response to negative pictures (vs. neutral pictures) but reduced activation in the dorsomedial prefrontal cortex (dMPFC) and AI‐dMPFC functional connectivity during emotion regulation compared to CON. Shift work was associated with increased motor activity during the most active 10‐h period, which then contributed to increased AI response to negative pictures. Our findings suggest that shift work may be associated with the neurobiological alterations of emotion regulation. Furthermore, increased motor activity may serve as a pathway through which shift work could contribute to neurobiological alterations associated with emotional regulation.

## 1 Introduction

Shift work follows non‐standard working hours (e.g., from 7:00 a.m. to 6:00 p.m.) that often misalign natural circadian rhythms, approximately a 24 h wake–sleep cycle (Torquati et al. 2019). Shift work is essential for providing adequate services and productivity in several workplaces, including healthcare, transportation and protective services (Brown et al. 2020). Given the important role of and increased demands on shift work in the diverse workplaces of modern societies, shift work has become more prevalent (Cheng and Drake 2018; Wright Jr. et al. 2013) and may contribute to elevating the risk of health problems. Indeed, shift work has been recognised as a risk factor for various health problems, such as sleep‐related (e.g., insomnia) and mood problems (e.g., depressed mood and anxiety) (Torquati et al. 2019). SW often experience sleep disturbance and circadian disruption (James et al. 2017; Kervezee et al. 2020) and show a greater prevalence of major depressive disorders than non‐SW (Ohayon and Hong 2006).

Emotion dysregulation, including heightened emotional reactivity and emotion regulation difficulties, is closely associated with sleep disturbance and circadian disruption (Chellappa 2020; Gruber and Cassoff 2014) and is a common feature of mood problems (Beauchaine and Cicchetti 2019). Healthy individuals and clinical samples who experience disrupted sleep and circadian rhythms often report heightened emotional reactivity to negative events and difficulty in regulating negative emotions (Mauss et al. 2013; O'Leary, Bylsma, et al. 2017; O'Leary, Small, et al. 2017; Palagini et al. 2022). Given that SW are vulnerable to sleep and circadian disturbances (James et al. 2017; Kervezee et al. 2020) and mood problems (James et al. 2017; Jagannath et al. 2013; Jones and Benca 2015), shift work may also be associated with emotion dysregulation.

Furthermore, emotional reactivity and regulation have been correlated with neural activation in the subcortical‐limbic (e.g., amygdala and insula) and prefrontal regions (e.g., lateral prefrontal cortex [LPFC] and medial prefrontal cortex [MPFC]) (Buhle et al. 2014; Lindquist et al. 2016; Morawetz et al. 2020). The amygdala and anterior insula (AI) are involved in a wide range of emotional processes, such as emotion recognition (threat), encoding emotional arousal and subjective emotional experiences (Pessoa and Adolphs 2010; Uddin et al. 2017). The LPFC is known to play a role in a broad range of cognitive mechanisms, such as executive function and cognitive control (Dixon et al. 2017), while the MPFC, especially the dorsomedial MPFC (dMPFC), serves as a region of emotional evaluation and regulation, as well as social cognition through connections with subcortical regions (Dixon et al. 2017; Eickhoff et al. 2016; Etkin et al. 2011). It is possible that SW may show neural alterations in regions implicated in emotional reactivity and regulation as indicators of emotion dysregulation. There are two possible reasons for this. First, there is evidence that disrupted sleep and circadian rhythms, which are problematic features of SW, are associated with neural alterations in the regions involved in emotional reactivity and regulation (Chellappa 2020; Gruber and Cassoff 2014). For example, sleep disturbance increases neural activation in response to negative stimuli in the amygdala (Yoo et al. 2007), but is associated with reduced activation in the dMPFC and dorsal anterior cingulate cortex (dACC) during emotion regulation (Klumpp et al. 2017; Minkel et al. 2012). Second, mood problems, which are considered adverse consequences of shift work, appear to be altered in brain regions implicated in emotion dysregulation (Diener et al. 2012; Kolesar et al. 2019; Rive et al. 2013). However, relatively little is known about the neural bases of emotion regulation in SW and whether mood, sleep and circadian rhythms are associated with neural alterations in emotion regulation.

This study aimed to investigate neural alterations in the regions implicated in emotional reactivity and regulation in SW and relationships between mood, sleep disturbance, circadian rhythms and neural alterations of emotional reactivity and regulation. Here, we recruited SW with a non‐standard shift schedule and controls who did not work shift schedules. Both SW and controls completed self‐reported measures of sleep disturbance and mood problems (visit 1), kept a sleep diary, and wore a wrist actigraphy device for 7 consecutive days. They also underwent one‐night polysomnography (visit 2) and were scanned while they were performing the emotion regulation task (visit 3). More specifically, we have attempted to achieve three goals. First, we elucidated the neural basis of emotional dysregulation based on emotional reactivity and regulation in SW compared to controls. Second, we examined the relationships between sleep disturbances, 24‐h rest‐activity rhythm (RAR) variables assessed by actigraphy, mood problems, and neural measures of emotional reactivity and regulation. We also examined whether the relationships between those factors differed between SW and controls. Third, based on the extant literature (Chellappa 2020; Gruber and Cassoff 2014), we further tested mediation models to examine whether shift work increases sleep and 24‐h RAR disturbances, which may then contribute to neural alterations in the regions implicated in emotion regulation.

We hypothesised that relative to controls, SW would show greater neural activation in response to negative pictures in the subcortical‐limbic regions (e.g., amygdala and AI), while they would show lower activation in the prefrontal regions (e.g., LPFC and MPFC) and functional connectivity between the prefrontal and subcortical‐limbic regions during emotion regulation. Second, measures of sleep, 24‐h RAR, and mood problems would be significantly correlated with the neural alterations of emotional reactivity and regulation, as we expect those correlations to also differ between SW and controls. Third, disrupted sleep and 24‐h RAR would play a mediating role in linking shift work and the neural alterations of emotion regulation.

## 2 Methods

### 2.1 Participants

One hundred twenty‐nine adults, including 65 SW and 64 controls (CON) who were not involved in shift work, were initially recruited through advertisements at the Seoul National University Hospital and Samsung Medical Center. SW were defined as individuals who worked outside of typical daytime working hours (9 a.m.–6 p.m.) by changing or rotating their shifts, such as three‐shift rotations of 8 h each, for example. Our study only included individuals who worked as SW for more than 6 months. Approximately 70% of SW was nurses and the remainder worked as medical staff, security personnel, engineers and unspecified jobs. The control group included individuals who did not engage in shift work and worked within typical daytime working hours. CON were included if they did not experience any sleep disturbances and typically slept at night. Those participants had various types of occupations, including office clerks, counsellors and teachers. Both groups included adults between the ages of 18 and 65 years. Participants in both SW and CON groups were excluded if they had a history of serious medical or neurological illness; common sleep disorders such as obstructive sleep apnoea (OSA, apnea‐hypopnea index [AHI] score ≥ 15) and periodic limb movement disorder (PLMD, periodic limb movement index [PLMI] score ≥ 15); Axis I psychiatric disorders other than a shift work type of circadian rhythm sleep disorder (SWD) (as defined by the Diagnostic and Statistical Manual of Mental Disorders‐IV); sleep disorders other than SWD (based on the International Classification of Sleep Disorders‐3 criteria); pregnancy; and any contraindication (e.g., presence of metal objects, including pacemakers and dental implants in the body) for magnetic resonance imaging (MRI) scans.

### 2.2 Procedure

This study was approved by the Institutional Review Boards of Seoul National University Hospital and Samsung Medical Center. All participants provided written informed consent prior to participating in the study. The participants made three visits. There were approximately 1‐ or 2‐month intervals between each visit due to the participants' schedules and availability for polysomnography and MRI scans (see Figure S1 for the details). SW was not allowed to make any visits on the day immediately after their night shifts.

During the first visit, all participants were assessed using the Structured Clinical Interview for the DSM‐IV (First et al. 1997) and were asked to complete questionnaires assessing their clinical characteristics. Through SCID, participants with psychiatric disorders were screened. They were also asked to complete questionnaires assessing their sleep (e.g., sleep quality and insomnia) and mood problems (e.g., depressive symptoms and anxiety). When they left their first visit, they were provided with a sleep diary and Actiwatch 2 to measure their sleep patterns and motor activity for the next 7 consecutive days. They were given instructions on how to keep a sleep diary and manage the Actiwatch wearing on their non‐dominant arm and return it during the second visit. During the second visit, the participants underwent nocturnal laboratory polysomnography (PSG; Profusion PSG3; Compumedics, Abbotsford, Victoria, Australia and Embla N7000 system; Embla Systems, Broomfield, CO, USA) to screen out individuals with common sleep disorders such as OSA and PLMD. During their third visit, the participants underwent an fMRI assessment while performing an emotion regulation task. Participants were given task instructions and practiced the task prior to the fMRI assessment.

### 2.3 Self‐Report Measures of Mood and Sleep Problems

Self‐reported questionnaires were used to measure subjective mood and sleep problems. The Beck Depression Inventory (BDI) and Beck Anxiety Inventory (BAI) were used to assess depressive symptom severity and anxiety symptoms, respectively (Beck et al. 1988; Beck and Steer 1984). Sleep quality was assessed using the Pittsburgh Sleep Quality Index (PSQI) (Buysse et al. 1989). The Insomnia Severity Index (ISI) was used to measure the features and severity of insomnia during the past month (Morin et al. 2011). Daytime sleepiness was assessed using the Epworth Sleepiness Scale (ESS) (Johns 1991, 1992). More detailed information on these self‐report measures is provided in the Supporting Information.

### 2.4 Sleep Diary Measures of Sleep Variables

A sleep diary was used to estimate the subjective aspects of the sleep patterns. Participants were asked to complete the diary within 10 min after awaking every morning for 7 consecutive days. Such daily sleep diaries provide information on night‐to‐night sleep experiences and variations. The four sleep parameters extracted for this study included total sleep time (TST), sleep efficiency (SE), sleep onset latency (SOL) and wake time after sleep onset (WASO).

### 2.5 Actigraphy Measures of Sleep and 24‐h RAR Variables

Actigraphy has been widely used to objectively measure sleep patterns and motor activity for days using a noninvasive accelerometer (Ancoli‐Israel et al. 2003). Our participants were provided with a portable Actiwatch 2 (Philips Respironics; Murrysville, PA, USA) and were instructed to wear it on their non‐dominant wrist for seven continuous days. Four sleep variables, TST, SE, SOL and WASO, were estimated. A cosinor analysis estimated four 24‐h RAR variables: the midline estimated statistic of rhythm (MESOR), acrophase, amplitude and *F*‐statistic. Nonparametric features of 24‐h RAR variables were also estimated: intradaily variability (IV), interdaily stability (IS), the most active 10‐h period (M10), the least active 5‐h period (L5) and relative amplitude (RA). More detailed information on actigraphy data analysis and 24‐h RAR variables is provided in the Supporting Information.

### 2.6 fMRI Emotion Regulation Task

As in the previous studies (Lee et al. 2022, 2021; New et al. 2009), each trial began with the presentation of a fixation cross for 1 s, followed by negative socio‐affective pictures or neutral pictures (4 s). An emotion regulation cue was superimposed on the centre of the picture for 1 s, and the picture continued to be displayed while emotions were regulated (i.e., suppressed or maintained) for the next 7 s. Subsequently, participants were asked to rate the intensity of their emotions based on a rating scale (1 = neutral, 2 = negative, 3 = very negative) presented for 4 s, followed by a fixation dot (4 s). In the “suppress” condition, participants were instructed to suppress their emotional response to negative socio‐affective pictures (i.e., try not to feel any emotions). In the “maintain” condition, participants were asked to maintain their responses to negative socio‐affective and neutral pictures. The example trials for negative and neutral image conditions are depicted in Figure S2.

**FIGURE 1:**
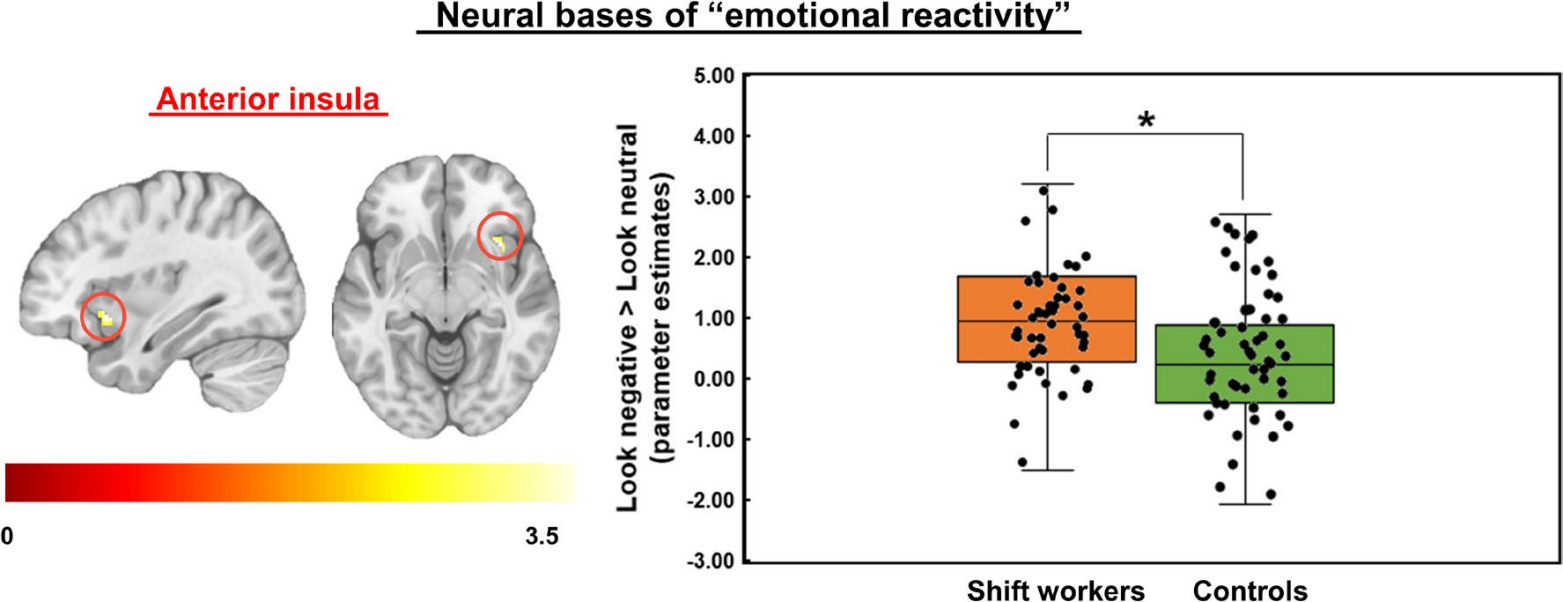
Group differences in the neural basis of emotional reactivity. Shift workers showed greater anterior insula activation in response to negative pictures (vs. neutral pictures) compared to controls. *Corrected *p* < 0.05.

In total, 36 negative socio‐affective pictures (18 pictures per each emotion regulation condition) and 18 neutral pictures (only for the “maintain” condition) were used. Neutral pictures were not used for the “suppress” condition. For example, negative stimuli induce negatively valenced emotional responses, and neutral stimuli are rated as neutral. Negative pictures depicted negatively valenced social situations, such as a person suffering from physical abuse by other people, and neutral pictures included neutral social situations, such as people walking together. These negative and neutral pictures, which were taken from the Korean Social Affective Visual Stimuli (K‐SAVS) (Seok et al. 2018), were validated. The K‐SAVS is a set of normative socio‐emotional stimuli for experimental studies of emotional and social processing. To select negative and neutral images from the K‐SAVS for this study, we conducted a rating study in which participants were asked to rate each image on the dimensions of valence and arousal using the modified Self‐Assessment Manikin (SAM) (Kang et al. 2011). The modified SAM used a 5‐point rating scale, ranging from a frowning, unhappy figure (1) to a smiling, happy figure (5) for the valence dimension; and from a relaxed, sleepy figure (1) to an excited, wide‐eyed figure (5). Mean (SD) valence and arousal ratings for negative images were 1.31 (0.26) and 3.59 (0.56), respectively, and for neutral images were 2.57 (0.39) and 1.58 (0.56), respectively. Those differences in valence and arousal ratings between negative and neutral images were statistically significant.

### 2.7 fMRI Data Acquisition and Analysis

The fMRI assessment was conducted during daytime, between 10 a.m. and 4 p.m. Given that shift work was associated with daytime sleepiness (Waage et al. 2014), we attempted to ensure that the participants were alert enough and wakeful to engage in the regulation task. Participants completed the Stanford Sleepiness Scale (SSS) (Hoddes et al. 1972) to assess their subjective sleepiness right before entering the scanner. The SSS scale ranged from 1 (feeling active and vital, alert, wide awake, thus least sleepiness) to 7 (sleep onset soon, thus most sleepiness). Most participants reported scores between 1 and 2. Participants who self‐scored 2 (e.g., functioning at a high level, but not fully alert) were encouraged to do some activity, such as walking around the waiting room and going to the restroom, until their score dropped to 1.

The fMRI data were collected with a 3 T whole‐body Tim Trio scanner (Siemens AG, Erlangen, Germany) using a 12‐channel birdcage head coil and an interleaved T2*‐weighted echo planar imaging sequence. High‐resolution structural images were acquired using a T1‐weighted 3D gradient‐echo pulse sequence with magnetization‐prepared rapid gradient‐echo sequencing. fMRI data were preprocessed and analysed using SPM12 (Wellcome Trust Centre for Neuroimaging, London, UK). Exploratory voxel‐wise whole‐brain *t*‐tests were used to identify brain regions exhibiting group differences (SW vs. CON) in emotional reactivity (“looking at negative pictures” vs. “looking at neutral pictures” contrast) and emotion regulation (“suppressing emotions” vs. “looking at negative pictures” contrast). A generalised psychophysiological interaction (gPPI) analysis (McLaren et al. 2012) using the CONN connectivity toolbox in SPM 12 (Whitfield‐Gabrieli and Nieto‐Castanon 2012) was performed to examine task‐dependent functional connectivity between the seed regions (i.e., functionally identified regions of interest [ROI] from the whole brain analyses [e.g., subcortical‐limbic and prefrontal regions]). Further information on fMRI data acquisition and analysis is provided in the Supporting Information (see Supporting Information methods section: fMRI data analysis).

### 2.8 Statistical Analysis

Statistical analyses were conducted using the SPSS software (version 25.0; SPSS Inc., Chicago, IL, USA). Independent‐sample *t*‐tests and multivariate analysis of variance were performed to test for group differences in demographic characteristics and mood, sleep and 24‐h RAR variables. Chi‐square tests were used to assess group differences in the categorical variables. Multiple regression analyses were conducted to explore whether mood, sleep and 24‐h RAR features were associated with neural activation and functional connectivity in our ROIs (e.g., amygdala, AI and PFC regions) and to examine whether the relationships differed between SW and CON. Furthermore, the PROCESS macro (Hayes 2012) was used to examine the indirect effects of shift work on the neural substrates of emotion dysregulation via sleep and 24‐h RAR. Further information on statistical analysis is provided in the Supporting Information (see Supporting Information methods section: Statistical analysis).

## 3 Results

### 3.1 Demographic, Mood, Sleep and 24‐h RAR Characteristics

As mentioned, 126 adult participants were initially recruited. Twenty participants were excluded because of sleep disorders (*N* = 2 [2 CON with AHI scores ≥ 15]), T1 structural imaging problems (e.g., tumour) (*N* = 2 [1 SW and 1 CON]), task‐related errors (*N* = 6 [3 SW and 3 CON]), excessive head motion (*n* = 4 [2 SW and 2 CON]), or poor image quality (*n* = 7 [3 SW and 4 CON]). Thus, the final sample included 56 SW and 52 CON.

The demographic characteristics and questionnaire scores are presented in Table 1. As shown in Table 1, significant group differences were found in one actigraphy‐assessed sleep variable and all 24‐h RAR variables, except for acrophase. Relative to CON, SW showed lower actigraphy‐estimated SE and lower values of some 24‐h RAR variables (e.g., *F*‐stat, IS, IV and RA) but higher values of 24‐h RAR variables (MESOR, M10 and L5). These significant group differences remained after correcting for multiple comparisons (see Table 1). However, no significant group differences were found in any of the self‐reported measures of mood (i.e., BDI and BAI), subjective sleep variables assessed by self‐reported measures (e.g., PSQI and ESS) and the sleep diary, and other actigraphy‐assessed sleep variables. Given that we used the PSG to screen out common sleep disorders, we reported the PSG characteristics of the two groups in the Supporting Information. No significant group differences were observed in any PSG variables (see Table S1).

**TABLE 1:** Demographic, clinical, sleep and 24‐h RAR characteristics of shift workers and controls.

All participants, *n* = 108, M ± SD, median (IQR)				
Variable	Shift workers, *n* = 56	Controls, *n* = 52	Test	*p*
Demographic information				
Age, years	30.80 ± 6.94	31.69 ± 7.86	*t* = 0.62	0.534
Female, *n* (%)	42 (75.0)	36 (69.2)	*χ* ^*2*^ = 0.45	0.504
Duration of shift work exposure, months	63.91 ± 53.77	—	—	—
Self‐related mood problems				
BDI	8.2 ± 6.55, 5.5 (9.25)	6.71 ± 6.44, 6.0 (9.50)	*F* = 1.89	0.172
BAI	6.93 ± 6.57, 4.5 (9.00)	8.20 ± 7.10, 7.0 (9.50)	*F* = 0.58	0.448
Self‐reported sleep problems				
PSQI	6.70 ± 2.74 (6.00, 5.00)	6.06 ± 3.44 (5.00, 4.00)	*F* = 0.94	0.335
ISI	9.45 ± 5.07 (9.00, 7.75)	8.62 ± 6.08 (8.50, 8.75)	*F* = 0.53	0.470
ESS	9.25 ± 3.93 (9.00, 5.00)	7.85 ± 3.81 (7.50, 6.00)	*F* = 3.11	0.081
Sleep diary				
TST, minutes	401.29 ± 62.14 (418.00, 85.50)	408.48 ± 55.78 (407.00, 61.75)	*F* = 0.59	0.446
SE, %	86.13 ± 6.93 (86.76, 9.45)	83.75 ± 9.74 (87.06, 16.46)	*F* = 1.23	0.270
WASO, minutes	31.25 ± 20.21 (29.50, 25.75)	29.54 ± 21.99 (20.00, 29.25)	*F* = 0.03	0.872
SOL, minutes	18.93 ± 13.13 (14.00, 15.25)	14.69 ± 11.54 (11.00, 12.75)	*F* = 2.85	0.095
Actigraphy: Sleep variables				
TST, minutes	401.87 ± 50.26 (403.50, 64.00)	418.21 ± 61.12 (426.00, 100.00)	*F* = 1.21	0.275
SE, %	77.29 ± 7.28 (79.28, 9.32)	81.63 ± 4.75 (81.50, 4.52)	*F* = 5.85	**< 0.05**
WASO, minutes	51.83 ± 20.72 (45.75, 25.15)	43.97 ± 18.47 (38.86, 24.78)	*F* = 2.61	0.111
SOL, minutes	35.81 ± 23.13 (28.83, 30.29)	33.77 ± 18.09 (31.36, 21.83)	*F* = 0.02	0.888
Actigraphy: 24‐h RAR				
MESOR	100.46 ± 19.45 (96.75, 29.83)	81.39 ± 24.16 (78.50, 29.60)	*F* = 13.80	**< 0.001**
Amplitude	50.08 ± 25.08 (49.00, 35.95)	59.90 ± 16.59 (56.90, 18.40)	*F* = 3.67	0.059
Acrophase	16.00 ± 3.44 (15.79, 3.41)	16.44 ± 1.28 (16.53, 1.90)	*F* = 0.44	0.508
F‐stat	1286.07 ± 1036.80 (1026.15, 1358.20)	1998.30 ± 1033.31 (1986.50, 1173.50)	*F* = 8.04	**< 0.01**
M10	132.00 ± 34.45 (123.78, 44.48)	110.82 ± 30.86 (108.21, 50.01)	*F* = 5.84	**< 0.05**
L5	34.00 ± 22.08 (31.35, 31.85)	7.03 ± 4.38 (5.89, 7.77)	*F* = 37.97	**< 0.001**
Interdaily stability	0.32 ± 0.15 (0.32, 0.19)	0.49 ± 0.11 (0.47, 0.15)	*F* = 28.69	**< 0.001**
Intradaily variability	0.71 ± 0.19 (0.72, 0.31)	0.90 ± 0.24 (0.92, 0.35)	*F* = 19.55	**< 0.001**
Relative amplitude	0.60 ± 0.23 (0.62, 0.37)	0.88 ± 0.07 (0.91, 0.12)	*F* = 38.93	**< 0.001**

*Note*: 30 of 52 controls were available for sleep diary and actigraphy data. Bold *p* values indicate the significant group differences in actigraphy‐estimated variables.

Abbreviations: BAI, Beck Anxiety Inventory; BDI, Beck Depression Inventory; ESS, Epworth Sleepiness Scale; F‐stat, F statistic; ISI, insomnia severity index; L5, least active 5‐h period; M10, most active 10‐h period; MESOR, midline estimated statistic of rhythm; PSQI, Pittsburgh Sleep Quality Index; RAR, rest‐activity rhythm; SOL, sleep onset latency; TST, Total Sleep Time; WASO, wake after sleep onset.

### 3.2 fMRI Results of the Emotion Regulation Task

#### 3.2.1 Behavioural Ratings While Performing Emotion Regulation Task

Participants in both the SW and CON groups reported that they felt more intense emotional responses (emotional reactivity) to negative pictures than to neutral pictures in the “maintain” condition. They also rated the emotional response to negative pictures as less intense after regulating compared to after maintaining responses to negative pictures. However, there were no significant group differences in emotional responses to negative pictures (vs. neutral pictures) and in the emotional intensity after regulating compared to after maintaining responses to negative pictures (for more detailed information, see Supporting Information results section and Figure S3).

#### 3.2.2 Neural Activation in Response to Negative Pictures (Emotional Reactivity)

A voxel‐based whole brain analysis revealed significant group differences (SW>CON) in neural activation in response to negative pictures (vs. neutral pictures) in the AI and bilateral visual regions (uncorrected *p* < 0.005) (see Table S2). Of these regions, a small volume correction (SVC) method limited to the AI ROI mask was applied to identify a cluster of activations within the AI (cluster‐defining threshold, *p* < 0.005; cluster size > 21 voxels to achieve an SVC corrected *p* < 0.05). There were two reasons for applying the SVC method to the AI regions: First, the AI was one of our ROIs. Second, the AI regions survived the cluster‐defining threshold, *p* < 0.005, but the cluster size of the AI was not big enough to survive after multiple comparison corrections based on cluster extent. In the AI cluster (*xyz* MNI coordinates = 34, 16, −8, 30 voxels, peak *t*‐value = 3.82) identified by the SVC, the SW displayed greater neural activation in response to negative pictures (vs. neutral pictures) than CON (Figure 1). However, no regions were found in which CON showed a greater neural response to negative pictures (vs. neutral pictures) than SW. Furthermore, inconsistent with our hypothesis, none of the voxels in the amygdala, which was another ROI, survived when the uncorrected *p* threshold (i.e., *p* < 0.005) was applied. Thus, the SVC did not apply to the amygdala region.

#### 3.2.3 Neural Activation and Functional Connectivity During Emotion Regulation

**FIGURE 2:**
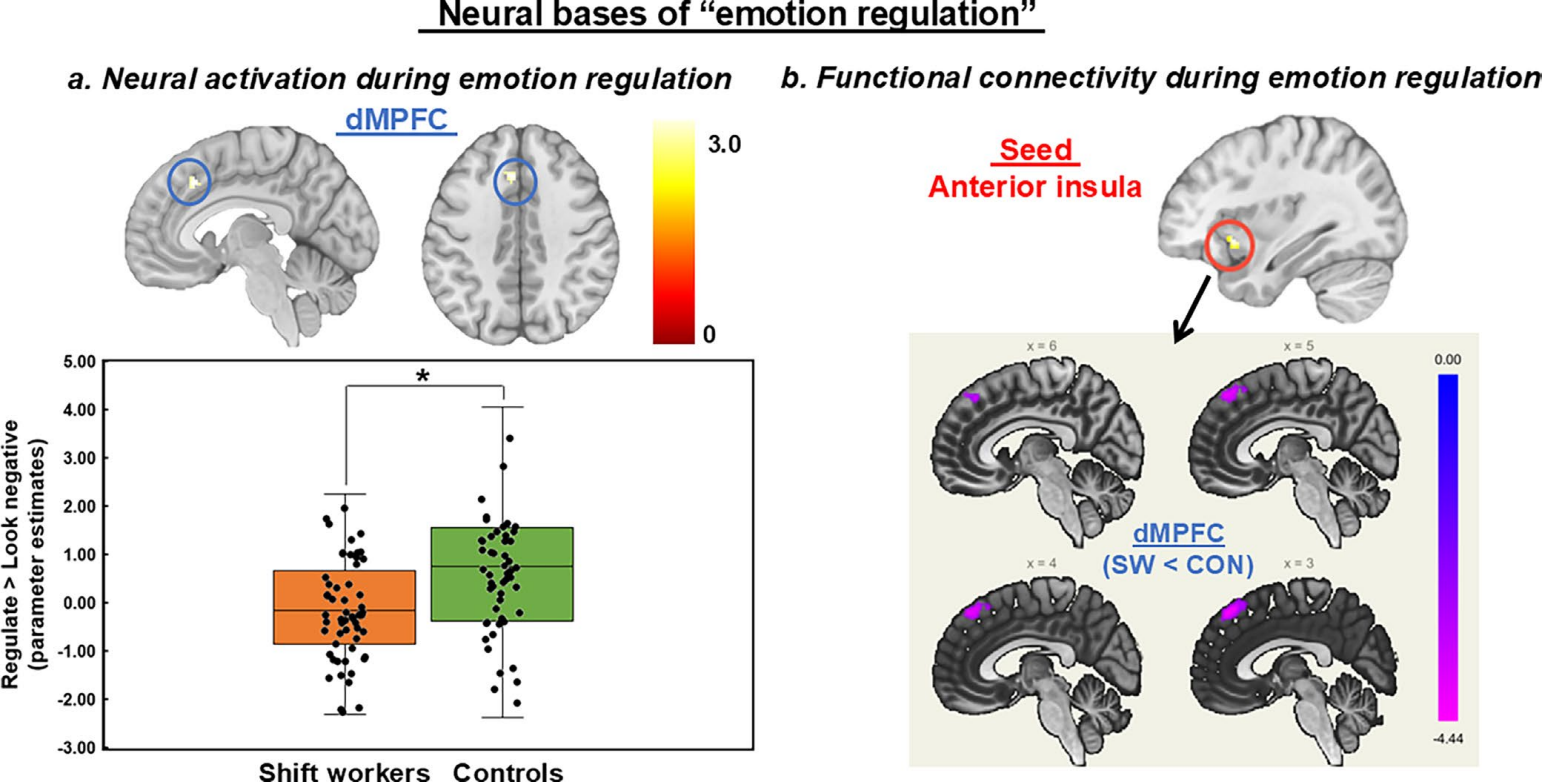
Group differences in the neural bases of emotion regulation. Shift workers showed lower dMPFC activation and lower functional connectivity between the anterior insula and dMPFC during regulating emotional response to negative pictures compared to controls. CON, controls; dMPFC, dorsomedial prefrontal cortex; SW, shift workers. *Corrected *p* < 0.05.

Results of the whole‐brain analysis showed significant group differences (SW<CON) during emotion regulation of negative emotions (vs. looking at negative pictures), including the superior middle frontal gyrus extending to the MPFC, posterior cingulate cortex (PCC), middle temporal gyrus and fusiform gyrus (Table S3; cluster‐defining threshold, *p* < 0.005; cluster size > 241 voxels to reach a corrected *p* < 0.05). Of these regions, a small volume correction (SVC) method using the dMPFC ROI mask was applied to identify a cluster of activations within the dMPFC implicated in emotion regulation (cluster‐defining threshold, *p* < 0.005; cluster size > 23 voxels to reach an SVC corrected *p* < 0.05). The SVC method was applied only to the dMPFC, which was our hypothesized ROI. The dMPFC was part of the large cluster, so the SVC was used to examine neural activation within the circumscribed dMPFC. In the dMPFC cluster (xyz MNI coordinates = −6, 24, 40, 28 voxels, peak *t*‐value = 3.13) identified by the SVC, SW displayed lower neural activation than CON during emotion regulation (Figure 2). No regions showed greater neural activation during emotion regulation in SW compared to CON.

Given that the AI and dMPFC were identified as regions showing group differences in emotional reactivity and regulation, we created functional masks using the AI and dMPFC clusters and conducted a gPPI analysis using these two seeds to elucidate their functional connectivity. Significant group differences (SW<CON) were found in functional connectivity between the right AI (seed) and the dMPFC (*xyz* MNI coordinates = 2, 40, 50, 203 voxels, cluster‐defining threshold = 0.005, FDR to reach a corrected *p* < 0.05). SW showed lower functional connectivity between the AI and dMPFC during the regulation of emotional response to negative pictures than CON (Figure 2). However, no significant group differences in functional connectivity were observed in the dMPFC seed.

### 3.3 Correlation Analysis Results

**FIGURE 3:**
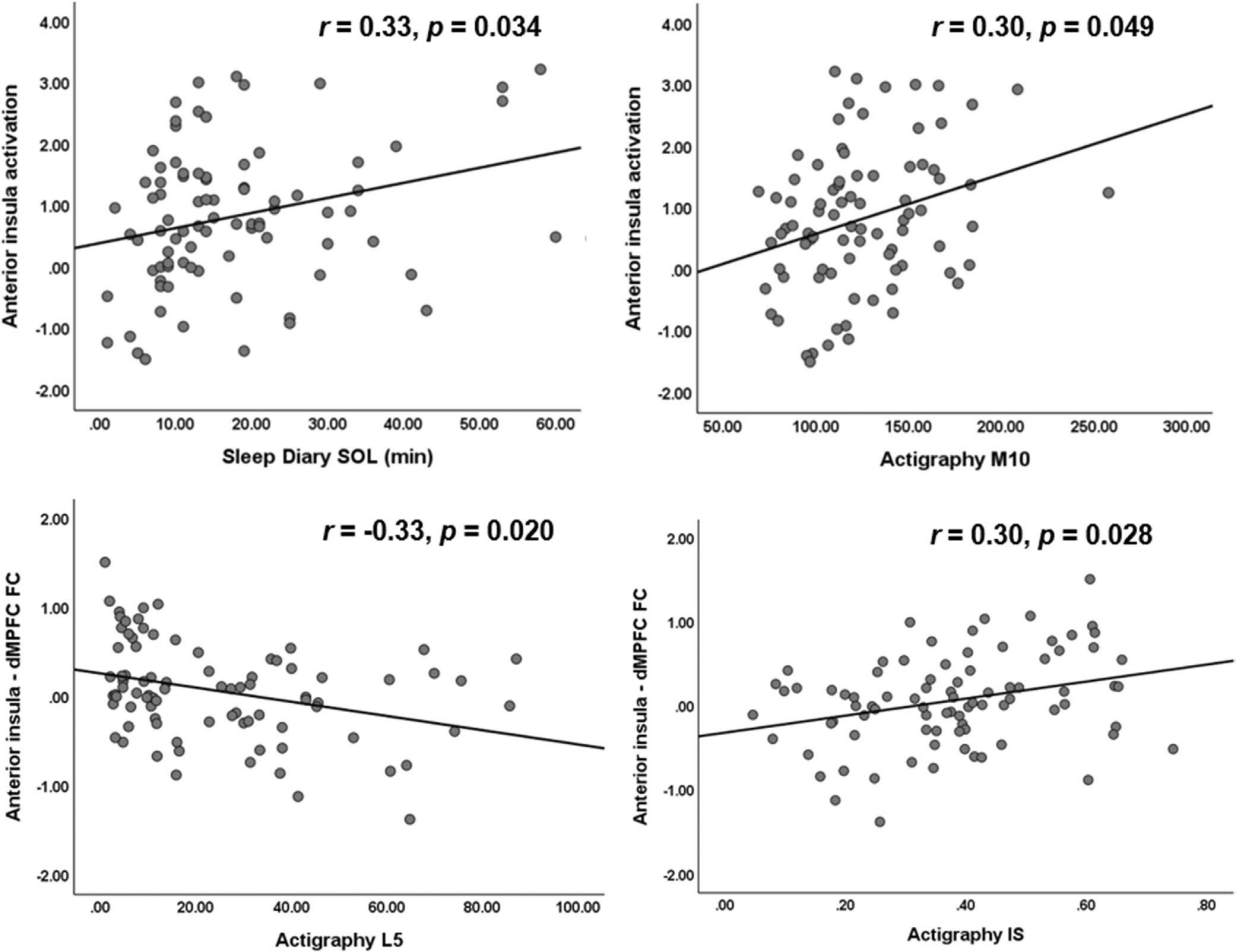
Correlations between sleep variables, circadian rest‐activity rhythm variables and neural measures. dMPFC, dorsomedial prefrontal cortex; FC, functional connectivity; IS, interdaily stability; L5, least active 5‐h period; M10, most active 10‐h period; *p*, false discovery rate‐corrected *p* value; SOL, sleep onset latency.

Sleep diary‐estimated SOL and actigraphy‐estimated M10 were positively correlated with AI activation in response to negative pictures (*r* = 0.33, *p* = 0.034 and *r* = 0.30, *p* = 0.049, respectively) (Figure 3). Actigraphy‐estimated L5 was negatively correlated with AI‐dMPFC functional connectivity during emotion regulation (*r* = −0.33, *p* = 0.020) (Figure 3). In contrast, actigraphy‐estimated RA and IS were positively associated with AI‐dMPFC functional connectivity during emotion regulation (*r* = 0.37, *p* = 0.011 and *r* = 0.30, *p* = 0.028 [Figure 3], respectively). These correlations did not differ between the SW and the CON. The results also remained significant after correcting for multiple comparisons and are summarised in Table S4. However, no significant correlations were found between self‐reported mood (i.e., BDI and BAI scores) and sleep problems (i.e., PSQI, ISI and ESS scores) and neural measures of emotional reactivity and regulation (*p*s > 0.17).

### 3.4 Mediation Analysis Results

**FIGURE 4:**
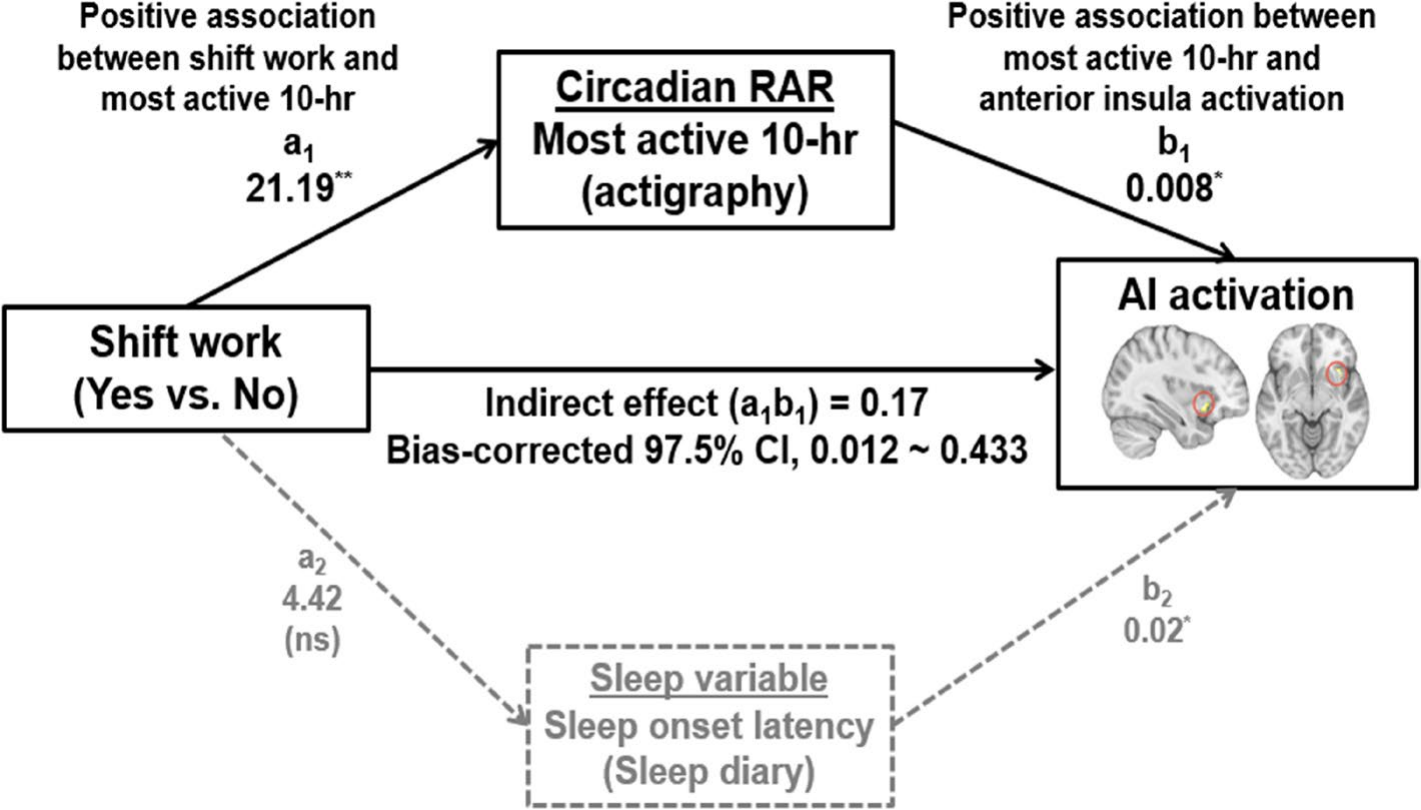
The mediation model describing the association between shift work (yes vs. no) and anterior insula activation in response to negative pictures (vs. neutral pictures) mediated by the most active 10‐h period, a 24‐h rest‐activity rhythm variable estimated by actigraphy, but not by subjective sleep onset latency estimated by sleep diary. Most active 10‐h, mean motor activity during the most active 10 h period; AI, anterior insula. *p < 0.05, **p < 0.01, ns = not significant.

Given the significant correlations between sleep, 24‐h RAR and neural measures (see Table S4), we examined whether sleep and 24‐h RAR variables mediated the association between shift work and emotional dysregulation (i.e., AI activation and AI‐dMPFC functional connectivity). We tested two mediation models with either AI activation or AI‐dMPFC functional connectivity as dependent variables, based on the correlation results. We found a significant indirect effect of shift work on AI activation in response to negative pictures (vs. neural pictures) through the 24‐h RAR variable, M10 (indirect effect = 0.17, 97.5% Bootstrap CI [0.012, 0.433]), but not through the sleep variable, SOL. The results are reported in Table S5 and Figure 4. However, 24‐h RAR variables, including L5 and IS, did not play significant mediating roles in the association between shift work and neural measures of emotion regulation (i.e., AI‐dMPFC functional connectivity during emotion regulation) (Table S6).

### 3.5 Additional Analysis Results: Controlling for Age and Gender

Although no group differences were observed depending on age and gender, due to the relatively wide range of age and high proportion of female participants, we conducted additional analyses including age and gender as covariates. The results reported above remained significant after controlling for age and gender. Thus, age and gender did not affect our main findings.

## 4 Discussion

This study aimed to elucidate the neural bases of emotion regulation based on emotional reactivity and regulation in SW. Furthermore, this study investigated the relationships between mood, sleep, 24‐h RAR and neural measures of emotion regulation. First, we found significant differences in actigraphy‐estimated sleep (i.e., lower sleep efficiency) and 24‐h RAR (e.g., greater motor activity and lower interdaily stability) in SW compared with CON. Second, SW exhibited increased AI activation in response to negative pictures (vs. neutral pictures) but reduced dMPFC activation and AI–dMPFC functional connectivity during emotion regulation compared with CON. Third, greater sleep disturbance (sleep diary‐estimated SOL) and motor activity (actigraphy‐estimated M10) were associated with increased AI activation in response to negative pictures. Greater 24‐h RAR disturbances, including L5, RA and IS, were also significantly associated with lower AI–dMPFC functional connectivity during emotion regulation. Fourth, greater motor activity during the most active 10‐h period mediated the association between shift work and increased AI activation in response to negative pictures.

As noted above, there were significant group differences in sleep and 24‐h RAR variables estimated using actigraphy, which is known to be an objective way to assess sleep and 24‐h RAR features. SW showed a greater sleep problem (i.e., lower actigraphy‐estimated sleep efficiency) than CON. Furthermore, SW were more likely to display disturbed 24‐h RAR, including greater motor activity (greater M10), less robust 24‐h rest‐activity rhythm (lower RA) and less restful sleep (greater L5), compared to CON. These results are somewhat similar to previous findings demonstrating sleep and circadian disturbances in SW (James et al. 2017; Kervezee et al. 2020). However, we did not find significant group differences in any of the subjective sleep measures assessed by questionnaires and sleep diary, and self‐reported mood problems. There are a few possible reasons for such insignificant findings. First, our SW were relatively young (mean age: 30.80 years) who may be less vulnerable to subjective sleep and mood problems. Second, they have been working with shift schedules not long enough (mean duration of shift work exposure: 63.91 months) to cause subjective sleep and mood problems. The reasons we suggested warrant further investigation.

Consistent with our hypothesis, relative to CON, SW demonstrated a greater neural response to negative pictures in the AI, which has been recognised as a central limbic region involved in a wide range of emotional processing, such as salient and negative emotional processing, interoceptive processing and physiological arousal (Zaki et al. 2012; Centanni et al. 2021). Thus, the increased AI response to negative pictures may reflect heightened emotional sensitivity to negative stimuli in SW. In contrast, during emotion regulation, SW showed lower activation in the dMPFC, which is one of the core regions involved in emotional regulation (Buhle et al. 2014; Morawetz et al. 2020) than CON. They also demonstrated lower AI and dMPFC functional connectivity during emotion regulation compared to CON. Lower dMPFC activation and AI–dMPFC functional connectivity may indicate difficulty in recruiting prefrontal activation or in downregulating emotional reactivity in SW. This interpretation may be in line with previous research showing reduced neural recruitment in PFC regions in clinical samples with emotion regulation difficulties (Beauchaine and Cicchetti 2019). However, given the limited understanding of the direction of any observed context‐modulated functional connectivity (Cisler et al. 2014), cautious results interpretation is needed. Taken together, these results may provide neurobiological evidence of emotion regulation difficulty (e.g., heightened emotional reactivity based on AI hyperactivation and emotion regulation difficulty represented by reduced dMPFC activation and AI‐dMPFC functional connectivity) in SW.

It is also important to note that no difference was observed between the groups in the ratings of emotional responses to negative pictures after regulation, indicating that similar to CON, SW may be able to subjectively regulate their emotions. SW, who was mostly healthcare professionals, may have a strong ability to regulate emotions or have knowledge of emotion regulation strategies, possibly due to emotion regulation training they have received. Despite the successful emotional regulation on the subjective level, SW's emotion regulation may be less effective on the neural level. In other words, SW may retain their emotion regulation capacity, but the brain areas involved in emotion regulation may experience alterations, potentially leading to emotional problems. Therefore, it is important to monitor brain function during emotion regulation in SW despite their ability to regulate emotions. To support this, future neuroimaging research assessing subjective emotion regulation ability using self‐report questionnaires (e.g., Difficulties in Emotion Regulation Scale and Emotion Regulation Questionnaire) is needed to understand the relationship between the neural correlates of emotion regulation, such as dMPFC activation and AI‐dMPFC functional connectivity, and subjective emotion regulation ability.

SW also showed lower activation in some posterior regions, including the PCC and visual cortex (e.g., fusiform and lingual gyri), which were outside our ROIs, during emotion regulation, compared to CON. This finding is consistent with the results of a recent meta‐analytic study demonstrating decreased PCC activation during emotion regulation in individuals with mood and anxiety disorders (Pico‐Perez et al. 2017). Given that the PCC serves as a diverse emotional and cognitive processing (e.g., subjective experience of emotion, self‐referential processing and monitoring internal and external information) (Foster et al. 2023; Raichle et al. 2001), lower PCC activation in SW may reflect reduced subjective emotional processing or monitoring cognitive processes, which are key elements for emotion regulation. The visual cortex has also been recognised as a significant region for processing emotional information (Kragel et al. 2019). The decreased activation in the visual cortices during emotion regulation may indicate that SW has deficient visual processing and visual attention to the negative pictures, consequently increasing the difficulty engaging in emotion regulation.

Furthermore, we found significant correlations between sleep disturbance, disturbed 24‐h RAR and the neural basis of emotional reactivity and regulation. First, longer sleep onset latency estimated by sleep diary and greater motor activity during the most active 10‐hperiod, which is usually the wake period, were associated with increased AI neural response to negative information. Prolonged sleep onset latency and motor activity are known to be associated with increased levels of cognitive and physiological arousal (Tang and Harvey 2004; Bonnet and Arand 2000). These results indicate that greater sleep disturbance and motor activity during wake times may be potential factors for emotional sensitivity. Second, greater alterations in 24‐h RAR (e.g., less restful sleep during sleep and poor synchronisation with the natural light–dark cycle) were significantly correlated with decreased dMPFC activity and AI‐dMPFC functional connectivity during emotion regulation. The literature suggests that 24‐h RAR disruption has adverse effects on emotion regulation and moon problems (Palagini et al. 2022; Walker 2nd et al. 2020). Our findings suggest that 24‐h RAR disruption may be closely related to difficulties in regulating negative emotions through AI‐dMPFC interactions. Taken together, sleep and 24‐h RAR disturbances may be important factors in heightened emotional reactivity and difficulties in emotion regulation.

Finally, we found that shift work was associated with increased motor activity during the most active 10‐h period, which then contributed to increased AI activation in response to negative information. This result partly supported the mediation model, which proposed that disrupted 24‐h RAR serves as a pathway through which shift work contributes to neural alteration in regions involved in emotion regulation. Increased motor activity in SW may indicate elevated occupational physical activity (i.e., physical activity on the job) or physical workload during shift work. This was consistent with previous research showing greater actigraphy‐estimated motor activity, such as fast walking and standing related to job demands, in hospital SW compared to non‐SW (Loef et al. 2018). Physical activity related to job demands may require sustained activation of SW' psychophysiological systems, potentially leading to increased physiological arousal (Bonnet and Arand 2000) and psychological distress (e.g., negative affect) (White et al. 2017). Furthermore, increased occupational physical activity may be an issue contributing to fatigue and burnout without enough time for recovery (Gupta et al. 2020). Thus, physiological arousal and psychological distress associated with occupational physical activity over time may contribute to alterations in the neural system involved in emotion regulation. Similarly, the physical activity paradox suggested that occupational physical activity has a negative impact on health (Gupta et al. 2020). Our results may provide evidence that occupational physical activity related to shift work impairs emotional health. To the best of our knowledge, this is one of the first empirical studies analysing the adverse effects of shift work on emotion regulation through disrupted 24‐h RAR. However, future research is necessary to confirm our findings by disentangling occupational physical activity from leisure‐related physical activity and measuring physiological arousal levels and psychological distress along with work‐related motor activity in SW.

This study had some limitations. First, the cross‐sectional study design limited our ability to infer causal effects of shift work on sleep and mood problems and neural alterations. Sampling bias may also be another issue associated with the difficulty in inferring causality because most SW in this study who were young adults and selected from healthcare professional occupations (e.g., nurses) may not represent the shift work population, consequently contributing to misleading causal inferences. For example, relatively young nurses may be more tolerable of shift work compared to SW with other occupations. Longitudinal studies with SW selected from diverse occupations could be better suited to understand temporal relationships between shift work and its outcomes. Second, although we could assume the shift schedules of nurses, approximately 70% of SW who participated in this study, we did not collect detailed information about shift work, such as shift schedules and social life, that could affect sleep, mood and 24‐h RAR. Thus, a larger sample size of SW may be needed to examine more specific effects of shift work on mood, sleep and 24‐h RAR after controlling for some factors such as shift schedules and social life. Third, given that the majority of SW participating in this study was healthcare professionals (e.g., nurses), our findings have limited generalizability to other shift‐working populations. However, the results can still be applicable to understand sleep problems, 24‐h RAR and neural alterations associated with emotion regulation in non‐healthcare SW as far as they work with non‐common shift schedules. As mentioned before, given that shift work has become more prevalent and diverse in modern societies, neuroimaging studies to investigate brain function and its association with other health problems are necessary with diverse shift work occupations, including firefighters, security guards and call/service centre representatives. Thus, future research should include diverse occupational shift working groups and consider potential confounding effects from different lifestyles as well as the social and environmental factors associated with diverse occupations. Fourth, given that participants in the control group had various types of occupations without reporting their specialties, we were unable to control for other factors, such as levels of job stress and job‐related activity, which may also affect sleep problems, mood and emotion regulation ability. Fifth, we collected sleep diaries and actigraphy for only 7 consecutive days, which may not be enough to distinguish sleep and 24‐h RAR characteristics after the day shift from after the night shift. Further research is needed to collect sleep diaries and actigraphy data for longer durations, such as more than 10 consecutive days. Furthermore, extending the duration of the sleep diary and the actigraphy monitoring may allow for a better understanding of the long‐term effects of shift work on sleep, circadian rhythm and emotion regulation. Sixth, individual differences in personality, resilience, lifestyle habits and pre‐existing health conditions or health status may affect our results on sleep problems, circadian rhythms and neural correlates of emotion regulation. However, the current study did not measure such individual differences, where the possible effects of individual differences on our results were not entirely controlled Seventh, we assessed sleep and mood problems using self‐report questionnaires, which might have generated response bias. Incorporating clinical interviews and additional objective measures may help to better understand sleep and mood problems in SW. Finally, we used the gPPI to examine task‐modulated functional connectivity because the gPPI has been widely used (Berboth and Morawetz 2021) and is known as an effective tool in detecting differences in functional connectivity between task conditions (Cisler et al. 2014; Di and Biswal 2019). However, when using the gPPI method for an event‐related fMRI design, which was employed in this study, there are some caveats that should be considered, such as the number of trials, interstimulus interval (ISI) and application of deconvolution (Di and Biswal 2019; Masharipov et al. 2024). Thus, to improve power or sensitivity in detecting task‐modulated functional connectivity even with subtle differences using the gPPI, future research should include a larger number of trials, the use of a more preferable ISI, and deconvolution of time‐series with the hemodynamic response function.

Despite these limitations, this is the first neuroimaging study to elucidate the neural basis of emotion regulation in SW and relationships between neural, mood, sleep problems and 24‐h RAR measures. Our findings may provide neuroscientific evidence that shift work could be associated with sleep and 24‐h RAR disturbances assessed by actigraphy and neural alterations in the regions implicated in emotional reactivity and emotion regulation. Such evidence suggests that attention should be paid to emotional and sleep health among SW, despite the absence of self‐reported mood and sleep problems. Furthermore, the mediation result indicates that increased motor activity during wake times may serve as a possible pathway through which shift work contributes to greater neural alteration in regions involved in negative emotional processing. Our findings have several practical implications. Since SW showed objectively measured sleep disturbance and 24‐h RAR disruption, providing education, such as sleep hygiene and light exposure, may promote SW to create favourable workplace and home environments for keeping good sleep and rest‐activity rhythm. Considering neural alterations associated with emotional reactivity and emotion regulation in SW, emotional regulation training (e.g., cognitive reappraisal, mindfulness and stress management) may help them prevent or intervene in their emotional problems.

## Author Contributions

**Kyung Hwa Lee:** conceptualization, formal analysis, writing – original draft, writing – review and editing, visualization, data curation, methodology. **Ha Young Lee:** data curation, project administration, writing – review and editing. **Jeong Eun Jeon:** data curation, project administration, writing – review and editing. **Mi Hyun Lee:** data curation, project administration, writing – review and editing. **Jooyoung Lee:** data curation, project administration, writing – review and editing. **Jiyoon Shin:** data curation, methodology, writing – review and editing. **Min Cheol Seo:** methodology, data curation, writing – review and editing. **Yu Jin Lee:** conceptualization, supervision, funding acquisition, investigation, writing – review and editing. **Seog Ju Kim:** conceptualization, supervision, investigation, funding acquisition, writing – review and editing.

## Disclosure

The authors have nothing to report.

## Conflicts of Interest

The authors declare no conflicts of interest.

## Supporting information


Data S1.

## Data Availability

The data that support the findings of this study are available from the corresponding authors upon reasonable request.
