# Supplementary material for: Twenty‐Four Hour Rest–Activity Rhythm Disturbances and Neural Alterations Associated With Emotion Regulation in Shift Workers
Source: J Sleep Res. 2025 Apr 3;34(5):e70052. doi: 10.1111/jsr.70052 (PMC12426705; doi:10.1111/jsr.70052)
Supplement: Supplementary file 1 — Data S1. [file JSR-34-e70052-s001.docx]

**Supplementary materials**

**24-hour rest-activity rhythm disturbances and neural alterations associated with emotion regulation in shift workers**

Kyung Hwa Lee^12^, Ha Young Lee^1^, Jeong Eun Jeon^1^, Mi Hyun Lee^13^, Jooyoung Lee^4^,

Jiyoon Shin^1^, Min Cheol Seo^1^, Yu Jin Lee^1*^, Seog Ju Kim^4*^

^1^Department of Psychiatry and Center for Sleep and Chronobiology, Seoul National University, College of Medicine and Hospital, Seoul, Republic of Korea

^2^Division of Child and Adolescent Psychiatry, Department of Psychiatry, Seoul National University Hospital, Seoul, Republic of Korea

^3^Seoul Top Class Clinic, Republic of Korea

^4^Department of Psychiatry, Sungkyunkwan University College of Medicine,

Samsung Medical Center, Seoul, Republic of Korea

^*^Corresponding authors

Seog Ju Kim, MD, PhD, Department of Psychiatry, Sungkyunkwan University College of Medicine, Samsung Medical Center, Seoul, Republic of Korea

Email: [ksj7126@skku.edu](mailto:ksj7126@skku.edu)

Yu Jin Lee, MD, PhD, Department of Psychiatry and Center for Sleep and Chronobiology, Seoul National University, College of Medicine and Hospital, Seoul, Republic of Korea

Email: [ewpsyche@snu.ac.kr](mailto:ewpsyche@snu.ac.kr)

**Supplementary Methods**

**Self-report measures of mood and sleep problems**

Self-reported questionnaires were used to measure subjective mood and sleep problems. The Beck Depression Inventory (BDI) has been widely used to assess depressive symptom severity during the past two weeks [1, 2]. It is a self-report scale with 21 items assessing various symptoms of depression, such as cognitive, emotional, physical, and motivational symptoms using a 4-point Likert scale. The Beck Anxiety Inventory (BAI) is a 21-item self-report questionnaire that assesses common symptoms of anxiety during the past week using a 4-point Likert scale [3, 4].

Sleep quality was assessed using the Pittsburgh Sleep Quality Index (PSQI) [5, 6], which was used to assess sleep disturbance and included 19 items regarding seven components (subjective sleep quality, sleep latency, sleep duration, habitual sleep efficiency, sleep disturbance, sleep medications, and daytime dysfunction) of sleep during the past month. The sum of the seven component scores reflects a PSQI global score of sleep quality, ranging from 0 to 21. Thus, higher PSQI global scores indicate greater sleep disturbance.

The Insomnia Severity Index (ISI) [7, 8] was used to measure the features and severity of insomnia during the past month based on seven items: difficulty falling asleep, difficulty maintaining sleep, problem waking up too early, satisfaction with current sleep patterns, interference with daily functioning, sleep problems noticed by others, and distress due to current sleep problems. Each item was rated on a 5-point Likert scale ranging from 0 (no problem) to 4 (severe problem), and possible total scores ranged from 0 to 28. Higher ISI scores indicate greater insomnia severity.

Daytime sleepiness was assessed using the Epworth Sleepiness Scale (ESS) [9, 10], consisting of 8 items describing situations in which individuals are likely to fall asleep. The ESS estimated the likelihood of falling asleep in these eight situations. Each item is rated on a scale ranging from 0 to 3, resulting in a total score ranging from 0 to 24. Higher ESS scores indicate higher daytime sleepiness.

**Actigraphy measures of sleep and circadian rest-activity rhythm (RAR) variables**

Actigraphy has been widely used to objectively measure sleep patterns and motor activity for days using a noninvasive accelerometer [11]. Our participants were provided with a portable Actiwatch 2 (Philips Respironics; Murrysville, PA, USA) and were instructed to wear it on their non-dominant wrist for seven continuous days. Actigraphy data were obtained in 30-sec epochs and analyzed using Actiware (version 6.0.9), which used a validated auto-algorithm to estimate the sleep variables. Trained investigators (H. Y. Lee and J. E. Jeon) cross-checked the actigraphy data with a daily recorded sleep diary and manually marked rest intervals (i.e., bedtime and wake time based on the sleep diary) in the Actiware software. Such conjugation between actigraphy data and sleep diaries helped us better estimate sleep variables. The excluded intervals (e.g., take-off) were also marked based on the sleep diary. Four sleep variables, TST, SE, SOL, and WASO, were estimated.

Furthermore, a cosinor analysis was conducted to estimate circadian variables based on the rest-activity patterns [11, 12]. For this analysis, a cosine curve representing 24-h or near 24-h periods was applied to estimate a model fit to raw actigraphy data using the least-squares method. This analysis estimated four circadian RAR variables: the midline estimated statistic of rhythm (MESOR, mean motor activity level of the fitted 24-h rhythm), acrophase (time of the peak activity during the day), amplitude (the difference between the peak value and the MESOR), and F-statistic (rhythm robustness). Given that rest-activity patterns do not always fit the cosine curve, a nonparametric analysis was also conducted using activity count data [13]. This analysis allowed us to capture the nonparametric features of circadian RAR variables: intradaily variability (IV, rhythm fragmentation during 24 h), interdaily stability (IS, synchronization to the natural light-dark cycle), the most active 10-h period (M10), the least active 5-h (L5), and relative amplitude (RA, the difference between M10 and L5, divided by their sum). Thus, nine circadian RAR variables were estimated from cosinor and nonparametric analyses.

**fMRI data analysis**

***fMRI data acquisition and preprocessing.*** The fMRI data were acquired with a 3 T whole-body Tim Trio scanner (Siemens AG) using a 12-channel birdcage head coil and interleaved T2*-weighted echo planar imaging (repetition time [TR] = 2,000 ms, echo time [TE] = 30 ms, flip angle = 90°, slice thickness = 4.0 mm, in-plane resolution = 3.4 × 3.4 mm, no gap, 33 axial slices, field of view [FOV] = 220 mm, 220 volumes). High-resolution structural images were acquired with a T1-weighted 3D gradient echo pulse sequence with magnetization-prepared rapid gradient-echo sequencing (TR = 1670 ms, TE = 1.89 ms, flip angle = 9°, slice thickness = 1.0 mm, in-plane resolution = 1.0 × 1.0 mm, FOV = 250 mm).

The fMRI data were preprocessed using SPM12 (Wellcome Trust Centre for Neuroimaging, London, UK). The data were slice time-corrected, motion-corrected, co-registered with the high-resolution structural image, spatially normalized to Montreal Neurological Institute (MNI) space, and smoothed using a 6-mm full-width at half-maximum Gaussian kernel. Co-registered and normalized fMRI data were visually inspected for quality control. Artifact Detection Tools (ART; <http://www.nitrc.org/projects/artifact_detect/>) was used to identify outlier volumes. Outliers with significant head motion for each participant were detected if those were greater than 2 mm composite motion or had larger global mean intensity (i.e., difference in global mean intensity across functional volumes > 3 SD). The outlier volumes did not exceed 15% of the total volumes in any participants, so none were excluded from the final analysis. The outliers were also entered into the first-level general linear model (GLM) as nuisance regressors to remove possible artifacts.

***First-level analysis.*** First-level GLM analyses were conducted for each participant. Five regressors pertaining to the presentation of pictures (two regressors: “looking at negative pictures” and “looking at neutral pictures”) and the emotion regulation phase (three regressors: “suppressing emotion”, “maintaining emotion”, and “maintaining neutral”) were entered into the model. Other regressors were also created, corresponding to the fixation cross, rating phase, and fixation dot, but these were of no interest. Regressors were defined based on boxcar functions that were convolved with the canonical hemodynamic response function. Six head motion parameters and outliers were included in each participant’s GLM model, to control for the effects of head motion and outliers.

Based on previous research [14], contrast images were defined to identify brain regions showing greater activation when looking at negative pictures compared to neutral pictures (emotional reactivity: looking at negative pictures vs. looking at neutral pictures contrast) and when suppressing the emotional response to negative pictures compared to looking at negative pictures (emotion regulation: suppressing negative emotion vs. looking at negative pictures contrast). These contrast images were submitted to the second-level group analysis.

***Second-level group analysis.*** We conducted whole brain analyses to determine regions showing group differences in neural activation, in association with emotional reactivity in the context of the “looking at negative pictures > look at neutral pictures” contrast, and in association with emotion regulation in the context of the “suppressing negative emotion > looking at negative pictures” contrast. Cluster-wise correction was performed in 3dClustSim, with smoothing estimated via AFNI’s 3dFWHMx with “acf” procedure ([https://afni.nimh,nih,gov](rewritten://b15b0733-d592-41bc-b9f7-bd56bd8ddd5b), version 18.3.16) [15], version 18.3.16). Cluster size was determined using 10,000 Monte Carlo simulations, second nearest neighbor (NN2) clustering, and a two-sided threshold. Both the cluster-defining threshold and cluster size necessary to achieve a cluster-wise corrected *p* < .05 are reported in the results section. Given that our regions of interest (e.g., limbic and prefrontal regions) were relatively large, we applied a small volume correction (SVC) to our anterior insula and prefrontal anatomical masks (using our ROIs as an inclusive mask) and calculated the minimum cluster size required for an SVC-corrected *p* < .05 and cluster-defining threshold *p* < .005 (uncorrected) using 3dClustSim (version 18.3.16; AFNI; <https://afni.nimh.nih.gov>) [15]. Thus, this SVC method helped us to examine anterior insula and prefrontal involvement during emotional reactivity and emotion regulation.

To further examine group differences in subcortical-prefrontal FC during emotion regulation, we performed gPPI [16] using the connectivity toolbox CONN in SPM 12 [17]. The deconvolved time-course extracted from the seed regions, which were functionally defined, were used as a physiological regressor. For example, the anterior insula and dorsomedial prefrontal cortex, functionally defined from the whole brain analysis (functional ROI) was used as a seed. The experimental conditions of emotion reactivity (e.g., looking at negative pictures) and emotion regulation (e.g., suppressing negative emotion) were used as psychological regressors. The interactions between the time-courses of our seeds and the experimental conditions were used as PPI regressors, which were convolved with the HRF. The CONN toolbox allowed us to create seed-to-voxel FC maps for each contrast (i.e., suppressing negative emotion vs. looking at negative pictures) and for each subject, and then to test group differences in FC. The results of the seed-to-voxel gPPI analysis were corrected for multiple comparisons at a false discovery rate (FDR) corrected threshold of *p* < .05.

**Statistical analysis**

Statistical analyses were conducted using the SPSS software (version 25.0; SPSS Inc., Chicago, IL, USA). Independent-sample *t*-test and multivariate analysis of variance were performed to test for group differences in demographic characteristics and mood, sleep, and circadian RAR variables. Chi-square tests were used to assess group differences in the categorical variables. Repeated measures ANOVAs were used to examine whether group differences in behavioral ratings were affected by the emotion regulation conditions. Correlation analyses were conducted to explore whether mood (e.g., depressive symptoms), sleep (e.g., PSQI, diary-estimated SOL, and actigraphy-estimated SOL), and circadian RAR features (e.g., MESOR and IV) were associated with neural activation (i.e., mean parameter estimates of the contrasts) and functional connectivity (i.e., mean parameter estimates of functional connectivity) extracted from regions demonstrating significant group differences. Skewed variables were log-transformed and used for correlation analyses. The Benjamini-Hochberg method with a false discovery rate (FDR) of 0.05 was applied to correct for multiple correlation tests [18]. Furthermore, the PROCESS macro [19] was used to examine the indirect effects of shift work on the neural substrates of emotion dysregulation via sleep and circadian RAR. Sleep and circadian RAR variables showing significant correlations with the neural substrates of emotion dysregulation were entered as mediators in the model. This analysis was conducted using bootstrapping (i.e., 95% bias-corrected bootstrap confidence intervals [CI] for the indirect effects based on 10,000 bootstrap resamples). Indirect effects were considered significant if the 97.5% bias-corrected CI did not include zero [20].

**Supplementary Results**

**Behavioral ratings**

As in previous study [21], we conducted two repeated measures ANOVAs on ratings after emotion regulation conditions.

**Responses to negative pictures vs. neutral pictures.** A group (SW vs. CON) X condition (maintaining responses to negative vs. neutral pictures) repeated measures ANOVA revealed a significant main effect of the condition, *F*(1, 103) = 736.48, *p* < 0.001, *η_p_^2^* = 0.88, indicating that negative pictures induced more intense emotional responses compared to neutral pictures in both SW and CON (Figure S1). There was no significant group X condition interaction (*p* > 0.78) (Figure S1).

**Suppressing vs. maintaining responses to negative pictures.** A group (SW vs. CON) X condition (suppressing vs. maintaining responses to negative pictures) repeated measure ANOVA revealed a significant main effect of the condition, *F*(1, 103) = 2.11, *p* < 0.001, *η_p_^2^* = 0.28) (Figure S1). Both SW and CON reported less intense emotional responses to negative pictures after regulating compared to after maintaining responses to negative pictures. This result indicated that our participants successfully regulated their emotional responses to negative pictures when they were instructed to regulate their emotional responses to negative pictures (Figure S1).

**Supplementary tables and figure**

**Table S1.** Polysomnographic characteristics of shift workers and controls

|  | All participants, n =108, M ± SD | | | |
| --- | --- | --- | --- | --- |
|  | Shift workers, n = 56 | Controls, n = 52 | Test | p value |
| Time in bed, min | 436.97 ± 36.47 | 452.21 ± 50.72 | *F* = 1.94 | 0.17 |
| Total sleep time, min | 394.55 ± 59.76 | 403.66 ± 58.28 | *F* = 0.63 | 0.43 |
| Sleep efficiency, % | 90.22 ± 10.92 | 89.34 ± 9.18 | *F* = 0.03 | 0.87 |
| WASO, min | 32.28 ± 45.43 | 38.01 ± 39.79 | *F* = 0.11 | 0.75 |
| Sleep latency, min | 8.67 ± 6.82 | 10.55 ± 10.74 | *F* = 1.80 | 0.18 |
| REM latency, min | 94.35 ± 52.93 | 108.57 ± 64.30 | *F* = 0.38 | 0.54 |
| Stage N1 sleep, % | 10.47 ± 7.49 | 11.34 ± 6.97 | *F* = 0.03 | 0.85 |
| Stage N2 sleep, % | 65.67 ± 9.89 | 61.28 ± 11.76 | *F* = 3.30 | 0.07 |
| Stage N3 sleep, % | 4.74 ± 6.70 | 6.93 ± 8.07 | *F* = 1.57 | 0.21 |
| REM sleep, % | 19.13 ± 6.75 | 20.46 ± 5.63 | *F* = 2.23 | 0.14 |
| AHI | 1.73 ± 2.69 | 1.77 ± 2.82 | *F* = 0.03 | 0.86 |
| PLMSI | 1.91 ± 4.96 | 2.16 ± 4.61 | *F* = 0.06 | 0.80 |

Note. min, minute; WASO, wake time after sleep onset; REM, rapid eye movement; AHI, apnea-hypopnea index; PLMSI, Periodic Limb Movement of Sleep Index

**Table S2.** Exploratory whole brain analysis to identify brain regions associated with emotional reactivity ("viewing negative pictures vs. viewing neutral pictures" contrast) (uncorrected *p* < 0.005, cluster size > 30)

|  |  |  |  | Number of voxels | Cluster size | MNI coordinates | | |  |
| --- | --- | --- | --- | --- | --- | --- | --- | --- | --- |
| Cluster | Region |  | H | in region | (voxels) | *x* | *y* | *z* | Peak T |
|  | *SW > CON when viewing negative pictures vs. viewing neutral pictures* | | | | | | | |  |
| 1 | Fusiform gyrus |  | R | 39 | 42 | 30 | -60 | -4 | 3.83 |
|  | Lingual gyrus |  | R | 3 |  |  |  |  |  |
| 2 | Insula |  | R | 30 | 36 | 34 | 16 | -8 | 3.82 |
|  | Putamen |  | R | 6 |  |  |  |  |  |
| 3 | Lingual gyrus |  | R | 25 | 40 | 10 | -68 | 6 | 3.28 |
|  | Calcarine gyrus |  | R | 15 |  |  |  |  |  |
| 4 | Lingual gyrus |  | L | 27 | 44 | -4 | -60 | 2 | 3.24 |
|  | Calcarine gyrus |  | L | 9 |  |  |  |  |  |
|  | Vermis |  | L | 8 |  |  |  |  |  |
|  |  |  |  |  |  |  |  |  |  |
|  | *SW < CON when viewing negative pictures vs. viewing neutral pictures* | | | | | | | |  |
|  | - |  |  |  |  |  |  |  |  |

Note. SW, shift workers; CON, controls

**Table S3.** Exploratory whole brain analysis to identify brain regions associated with emotion regulation (“suppressing negative emotion” vs. “viewing negative pictures” contrast) (cluster-defining threshold, *p* < 0.005; cluster size > 241 voxels to achieve a cluster-wise corrected *p* < 0.05)

|  |  |  |  | Number of voxels | Cluster size | MNI coordinates | | |  |
| --- | --- | --- | --- | --- | --- | --- | --- | --- | --- |
| Cluster | Region |  | H | in region | (voxels) | *x* | *y* | *z* | Peak T |
|  | *SW > CON while regulating negative emotions (vs. viewing negative pictures)* | | | | | | | | |
|  | - |  |  |  |  |  |  |  |  |
|  | *SW < CON while regulating negative emotions (vs. viewing negative pictures)* | | | | | | | | |
| 1 | Fusiform gyrus |  | L | 124 | 275 | -38 | -24 | -20 | 4.53 |
|  | Cerebellum |  | L | 103 |  |  |  |  |  |
|  | Lingual gyrus |  | L | 48 |  |  |  |  |  |
| 2 | Posterior cingulate cortex | | - | 226 | 258 | -10 | -40 | 28 | 4.41 |
|  | Mid cingulate cortex |  | - | 32 |  |  |  |  |  |
| 3 | Middle temporal gyrus |  | R | 124 | 251 | 48 | 4 | -22 | 4.4 |
|  | Temporal pole |  | R | 127 |  |  |  |  |  |
| 4 | Postcentral gyrus |  | R | 224 | 245 | 34 | -32 | 44 | 4.05 |
|  | Precentral gyrus/inferior parietal lobe |  |  | 21 |  |  |  |  |  |
| 5 | Superior frontal gyrus |  | R | 108 | 309 | 26 | -16 | 62 | 3.72 |
|  | Mid cingulate cortex |  | - | 135 |  |  |  |  |  |
|  | Precentral gyrus |  | R | 66 |  |  |  |  |  |

Note. SW, shift workers; CON, controls

**Table S4.** Correlations between sleep variables, circadian variables, and neural variables

| **Sleep variables** | **Neural variables** | *r* | FDR-corrected *p* |
| --- | --- | --- | --- |
|  | **Emotional reactivity** |  |  |
| Sleep Diary_SOL^*#^ | Anterior insula | 0.33 | 0.034 |
| **Circadian variables** | **Neural variables** | *r* |  |
|  | **Emotional reactivity** |  |  |
| Actigraphy_M10^*#^ | Anterior insula | 0.30 | 0.049 |
|  | **Emotion regulation** |  |  |
| Actigraphy_L5^#^ | Anterior insula-dMPFC functional connectivity | -0.33 | 0.020 |
| Actigraphy_RA^#^ | Anterior insula-dMPFC functional connectivity | 0.37 | 0.011 |
| Actigraphy_IS | Anterior insula-dMPFC functional connectivity | 0.30 | 0.028 |

Note. SOL, sleep onset latency; M10, most active 10-h period; L5, least active 5-h period; RA, relative amplitude; IS, interdaily stability; dMPFC, dorsomedial prefrontal cortex; FC, functional connectivity, FDR, false discovery rate using Benjamini–Hochberg correction for multiple tests

^*^ log-transformed

^#^ Scatter plots were shown in Figure 3

**Table S5.** A summary of multiple mediation model for shift work, circadian rhythm and sleep variables and AI activation

| Independent variable | Multiple mediators | Dependent variable | Effect of IV on M | Effect of M on DV | Direct effect | **Indirect effect** | 97.5 % CI | |
| --- | --- | --- | --- | --- | --- | --- | --- | --- |
|  |  |  | a_i_ | b_i_ | c' | (a_i_ x b_i_) | LL | UL |
| Shift work  (Yes vs. No) | **1. Most active 10-hr**  **(actigraphy)** | Anterior insula (negative > neutral pictures) | **21.19**  **(SE = 7.64)^**^** | **0.008**  **(SE = 0.004)^*^** | 0.31  (SE = 0.26) | **0.17**  **(SE = 0.09)^*^** | **0.012** | **0.433** |
|  | 2. SOL  (sleep diary) |  | 4.42  (SE = 2.90) | 0.02  (SE = 0.009)^*^ |  | 0.09  (SE = 0.08) | -0.052 | 0.318 |

Note. AI, anterior insula; IV, independent variable; DV, dependent variable; M, mediators; SE, standard error; CI, confidence interval; LL, lower limit; UL, upper limit; Most active 10-hr, most active10 hour period, a circadian variable estimated by actigraphy; SOL, sleep onset latency, a sleep variable estimated by sleep diary.

^*^ *p* < 0.05, ^**^ *p* < 0.01

**Table S6.** A summary of multiple mediation model for shift work, circadian rhythm variables and AI-dMPFC functional connectivity

| Independent variable | Multiple mediators | Dependent variable | Effect of IV on M | Effect of M on DV | Direct effect | **Indirect effect** | 97.5 % CI | |
| --- | --- | --- | --- | --- | --- | --- | --- | --- |
|  |  |  | a_i_ | b_i_ | c' | (a_i_ x b_i_) | LL | UL |
| Shift work  (Yes vs. No) | 1. L5  (actigraphy) | FC between the anterior insula and dMPFC during emotion regulation | 26.95  (SE = 4.15)^***^ | 0.005  (SE = 0.007) | -0.45  (SE = 0.14)^**^ | 0.14  (SE = 0.20) | -0.349 | 0.601 |
|  | 2. RA  (actigraphy) |  | -0.28  (SE = 0.04)^***^ | 0.84  (SE = 0.74) |  | -0.24  (SE = 0.21) | -0.778 | 0.221 |
|  | 3. IS  (actigraphy) |  | 0.17  (SE = 0.03)^***^ | -0.14  (SE = 0.59) |  | 0.02  (SE = 0.10) | -0.209 | 0.269 |

Note. AI, anterior insula; dMPFC, dorsomedial prefrontal cortex; FC, functional connectivity; IV, independent variable; DV, dependent variable; M, mediators; SE, standard error; CI, confidence interval; LL, lower limit; UL, upper limit; L5, least active 5-hour period; RA, relative amplitude; IS, interdaily stability

^**^ *p* < 0.01, ^***^ *p* < 0.001


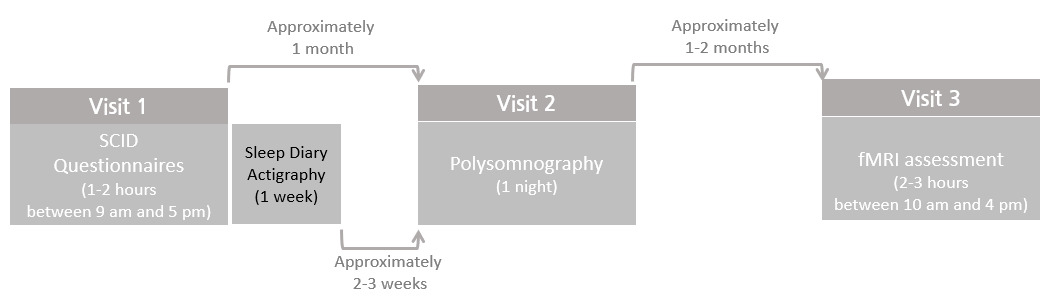


**Figure S1.** The timeline of our study


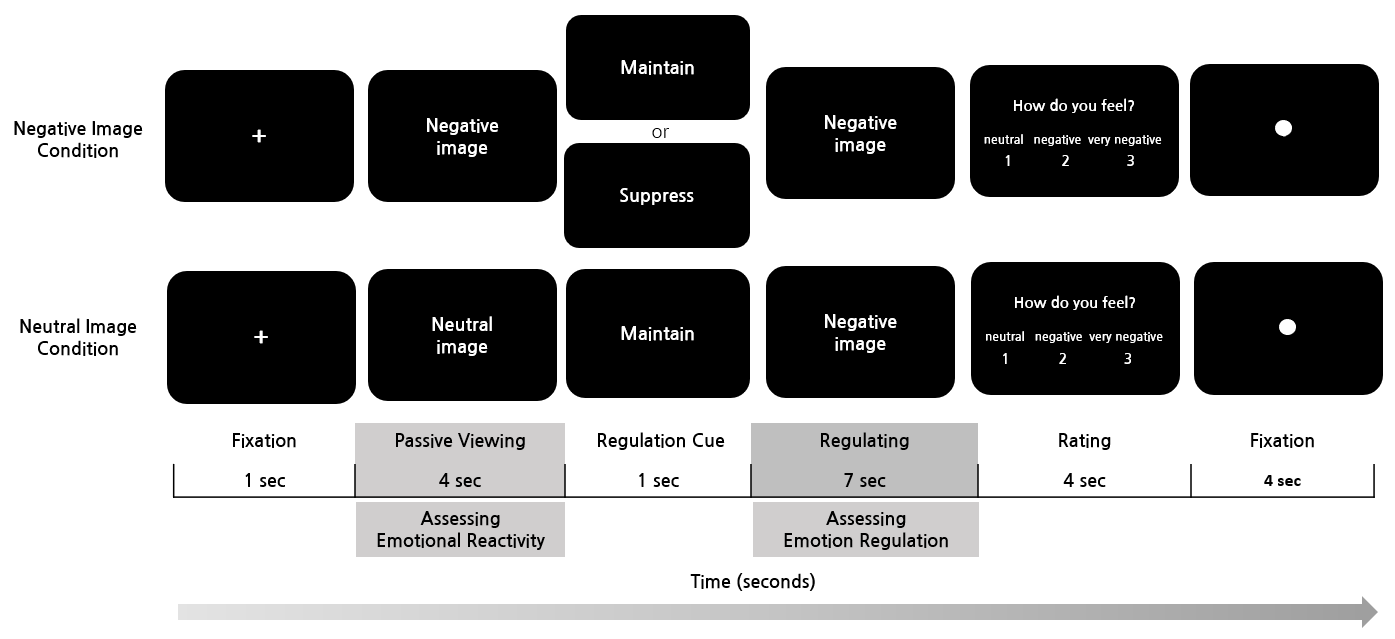


**Figure S2.** Depiction of example trials for negative and neutral image conditions in the fMRI emotion regulation task


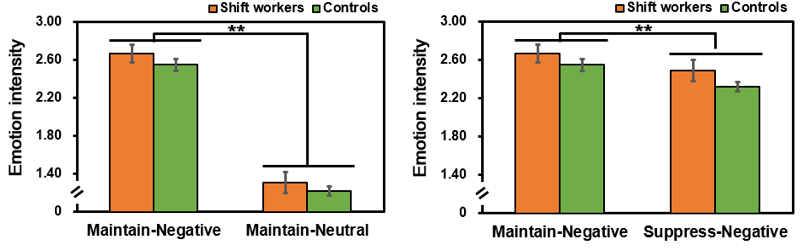


**Figure S3.** Subjective emotional intensity ratings after the emotion regulation trials. a) The significant main effect of the condition (more intense responses to negative pictures compared to neutral pictures): both SW and CON reported more intense responses after maintaining responses to negative pictures compared to maintaining responses to neural pictures. b) The significant main effect of the condition (reduced response to negative pictures after suppression): both SW and CON reported reduced responses to negative pictures after suppressing responses to negative pictures compared to maintaining responses to negative pictures.

^**^ *p* < 0.01

**References**

1. Beck AT, Steer RA. Internal Consistencies of the Original and Revised Beck Depression Inventory. J Clin Psychol. 1984; 40 (6): 1365-1367.

2. Rhee MY, Lee YH, Park SY, et al. A Standardization Study of Beck Depression Inventory 1 - Korean Version ( K - BDI ) : Reliability and Factor Analysis. The Korean Journal of Psychopathology. 1995; 4 (1): 77-95.

3. Beck AT, Brown G, Epstein N, Steer RA. An Inventory for Measuring Clinical Anxiety - Psychometric Properties. J Consult Clin Psych. 1988; 56 (6): 893-897.

4. Lee HK, Lee EH, Hwang ST, Hong SH, Kim JH. Psychometric properties of the Beck Anxiety Inventory in the community-dwelling sample of Korean adults. Korean J Clin Psychol. 2016; 35: 822-830.

5. Buysse DJ, Reynolds CF, 3rd, Monk TH, Berman SR, Kupfer DJ. The Pittsburgh Sleep Quality Index: a new instrument for psychiatric practice and research. Psychiatry Res. 1989; 28 (2): 193-213.

6. Sohn SI, Kim DH, Lee MY, Cho YW. The reliability and validity of the Korean version of the Pittsburgh Sleep Quality Index. Sleep Breath. 2012; 16 (3): 803-812.

7. Cho YW, Song ML, Morin CM. Validation of a Korean version of the insomnia severity index. J Clin Neurol. 2014; 10 (3): 210-215.

8. Morin CM, Belleville G, Belanger L, Ivers H. The Insomnia Severity Index: psychometric indicators to detect insomnia cases and evaluate treatment response. Sleep. 2011; 34 (5): 601-608.

9. Johns MW. A new method for measuring daytime sleepiness: the Epworth sleepiness scale. Sleep. 1991; 14 (6): 540-545.

10. Johns MW. Reliability and factor analysis of the Epworth Sleepiness Scale. Sleep. 1992; 15 (4): 376-381.

11. Ancoli-Israel S, Cole R, Alessi C, Chambers M, Moorcroft W, Pollak CP. The role of actigraphy in the study of sleep and circadian rhythms. Sleep. 2003; 26 (3): 342-392.

12. Hwang JY, Byun MS, Choe YM, et al. Moderating effect of APOE epsilon4 on the relationship between sleep-wake cycle and brain beta-amyloid. Neurology. 2018; 90 (13): e1167-e1173.

13. Mitchell JA, Quante M, Godbole S, et al. Variation in actigraphy-estimated rest-activity patterns by demographic factors. Chronobiol Int. 2017; 34 (8): 1042-1056.

14. Miller AB, McLaughlin KA, Busso DS, Brueck S, Peverill M, Sheridan MA. Neural Correlates of Emotion Regulation and Adolescent Suicidal Ideation. Biol Psychiatry Cogn Neurosci Neuroimaging. 2018; 3 (2): 125-132.

15. Cox RW. AFNI: software for analysis and visualization of functional magnetic resonance neuroimages. Comput Biomed Res. 1996; 29 (3): 162-173.

16. McLaren DG, Ries ML, Xu G, Johnson SC. A generalized form of context-dependent psychophysiological interactions (gPPI): a comparison to standard approaches. Neuroimage. 2012; 61 (4): 1277-1286.

17. Whitfield-Gabrieli S, Nieto-Castanon A. Conn: a functional connectivity toolbox for correlated and anticorrelated brain networks. Brain Connect. 2012; 2 (3): 125-141.

18. Benjamini Y, Hochberg Y. Controlling the False Discovery Rate - a Practical and Powerful Approach to Multiple Testing. J R Stat Soc B. 1995; 57 (1): 289-300.

19. Hayes AF. In. *PROCESS: A versatile computational tool for observed variable mediation, moderation, and conditional process modeling [White paper]*. Retrieved from <http://www.afhayes.com/public/process2012>, pdf 2012.

20. Preacher KJ, Hayes AF. Asymptotic and resampling strategies for assessing and comparing indirect effects in multiple mediator models. Behav Res Methods. 2008; 40 (3): 879-891.

21. McRae K, Gross JJ, Weber J, et al. The development of emotion regulation: an fMRI study of cognitive reappraisal in children, adolescents and young adults. Soc Cogn Affect Neurosci. 2012; 7 (1): 11-22.
